# Study on the applicability of pressurized physically activated carbon as an adsorbent in adsorption heat pumps†

**DOI:** 10.1039/d1ra08395c

**Published:** 2022-01-19

**Authors:** Hyeonseok Yi, Koji Nakabayashi, Seong-Ho Yoon, Jin Miyawaki

**Affiliations:** Interdisciplinary Graduate School of Engineering Sciences, Kyushu University 6-1 Kasuga-koen Kasuga Fukuoka 816-8580 Japan miyawaki@cm.kyushu-u.ac.jp +81 92 5837897 +81 92 5838857; Institute for Materials Chemistry and Engineering, Kyushu University 6-1 Kasuga-koen Kasuga Fukuoka 816-8580 Japan

## Abstract

Activated carbon is a suitable adsorbent for adsorption heat pumps (AHPs) with ethanol refrigerants. Although chemically activated carbon with highly developed pore structures exhibits good ethanol adsorption, the associated high production costs inhibit its practical application as an AHP adsorbent. Moreover, although physical activation can produce inexpensive activated carbon, the limited pore development limits the ethanol uptake. Recently, we developed a pressurized physical activation method that can produce activated carbon with a well-developed pore structure and characteristic pore size distribution. In this study, we investigated the applicability of the pressurized physically activated carbon as an adsorbent in activated carbon–ethanol AHP systems. Because of the large number of pressurization-induced pores of appropriate size, the pressurized physically activated carbon showed effective ethanol uptake comparable with that of chemically activated carbon on a weight basis. Furthermore, on a volume basis, the pressurized physically activated carbon, with a high bulk density, showed much higher effective ethanol uptake than chemically activated carbon. These results confirm the potential of the pressurized physically activated carbon as a relatively inexpensive high-performance adsorbent for AHP systems with ethanol refrigerants.

## Abstract 2

Thanks to the large number of pressurization-induced pores of appropriate size and the high bulk density, the pressurized physically activated carbon showed much higher effective ethanol uptake than chemically activated carbon.

## Graphical abstract

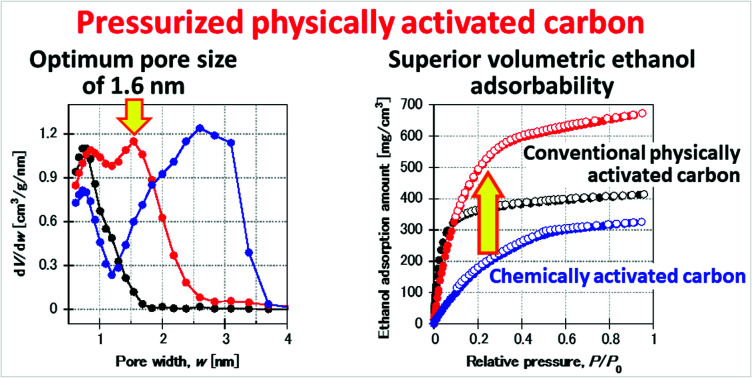

## 1. Introduction

Today, environmental friendliness, CO_2_ emission reduction, thermal energy efficiency, and thermal management are important global concerns. A heat pump is a system capable of cooling, heating, and freezing by pumping heat. Heat pumps are widely used in homes and industrial plants. According to their drive power, heat pumps are classified into vapor compression (compressor), thermoelectric element (Peltier element), and absorption/adsorption types. Among these, the adsorption heat pump (AHP) is commercially available as adsorption type refrigerators. These refrigerators are equipped with a porous adsorbent and operate based on the adsorption cooling principle proposed by Faraday in 1848.^1^ Unlike other types of heat pumps, AHPs require almost no electricity for their operation and produce minimal vibration and noise. Furthermore, AHPs can be driven by solar heat or industrial low-temperature waste heat, preventing CO_2_ emissions. Therefore, replacing existing heat pumps with AHPs can contribute to electric power saving and efficient waste thermal energy recycling. However, the large size of the existing AHP devices limits their widespread usage, necessitating the improvement of the performance of AHP systems.

Examples of AHP refrigerants include NH_3_, water, alcohol, and CO_2_,^2–4^ while porous materials such as silica gel, synthetic zeolite, and activated carbon are used as adsorbents.^5–7^ Synthetic zeolite–water is a commercial AHP adsorbent–refrigerant combination. Although water is inexpensive and safe, the zeolite–water pair is limited by low adsorption capacity, and water cannot obtain temperatures below ice point. Thus, activated carbon–ethanol has attracted increasing attention as an alternative pair. High ethanol adsorbability of activated carbons has been reported as summarized in Table 1. For example, owing to its well-developed pore structure, activated carbon has a saturated ethanol-uptake capacity of 1900 mg g^−1^.^10^ Saturated capacity is an important indicator of the maximum adsorption capacity of an adsorbent. Instead, “effective adsorption uptake” is used herein to denote the difference in adsorption uptake between adsorption and regeneration (desorption) conditions, as a critical index to assess the performance of AHP systems. This is because the AHP working pressure range is determined by the AHP operation temperatures (*i.e.*, the temperatures of cooling water, the surrounding environment, and regeneration) and does not include the overall relative pressure (*P*/*P*_0_) range of refrigerant (0–1). For example, the AHP *P*/*P*_0_ range was calculated as 0.1–0.3 of ethanol adsorption isotherms at 303 K under assumption of 283, 303, and 353 K for the cooling water, the surrounding environment, and regeneration temperatures, respectively. Under this condition, the maximum effective ethanol uptake occurred at a carbon pore slit width of 1.6 nm.^14^ Chemically activated carbon with an average pore width of 1.6 nm showed a high effective ethanol uptake of >700 mg g^−1^.^14^

**Table 1:** Recent studies on ethanol adsorbability of porous adsorbents toward AHP application

Adsorbent	Specific surface area [m^2^ g^−1^]	Total pore volume [cm^3^ g^−1^]	Uptake temperature [K]	Saturated ethanol uptake amount [mg g^−1^]	Reference
Petroleum coke-derived activated carbon (chemical activation)	3029	1.73	303	1230	8 and 9
Biomass-derived activated carbon (chemical activation)	2927	2.51	303	1900	10
Ethylene tar pitch-derived activated carbon fiber (physical activation)	1471	0.74	298	580	11
Metal–organic framework (chromium based)	Not provided	Not provided	298	1200	12
Metal–organic framework (chromium based)	3318	2.02	303	1100	13

However, conventional physical activation methods using atmospheric pressure CO_2_ or steam cannot easily realize activated carbon with a large number of 1.6 nm micropores. In contrast, chemical activation using KOH or NaOH as the activation agent can introduce a large number of micropores and mesopores; however, the required post-treatment step of acid-washing and anti-corrosion equipment increase production costs. Thus, we recently developed a pressurized physical activation method for activated carbon production.^15^ The obtained activated carbon showed a high degree of pore development and characteristic pore size distribution, with a peak pore width of approximately 1.6 nm.

In this study, we investigated the applicability of pressurized physically activated carbon in AHP systems using ethanol refrigerant.

## 2. Experimental

### 2.1 Sample preparation

We used a spherical 8 μm-diameter phenol resin (BEAPS series; ASAHI YUKIZAI CORPORATION, Japan) as the raw material. Carbonization was performed at 873 K (600 °C) and held for 1 h under a N_2_ flow rate of 200 mL min^−1^ to obtain a spherical carbonized product (C6). C6 was activated at 1173 K under CO_2_ pressures of 0.1 MPa for 90 min and 1.0 MPa for 5 min to obtain atmospheric pressure and pressurized physically activated carbons, respectively. During chemical activation, a mixture of C6 and KOH (1 : 6 by weight) was heated to 1173 K and held for 1 h under N_2_ flow. Afterward, washing, filtering, and drying were performed to obtain chemically activated carbon. Activated carbons obtained by atmospheric pressure physical activation (CO_2_-activated carbon), pressurized physical activation (pressurized CO_2_-activated carbon), and chemical activation (KOH-activated carbon) are abbreviated as PAC, PPAC, and CAC, respectively. Activation yield was calculated as follows:
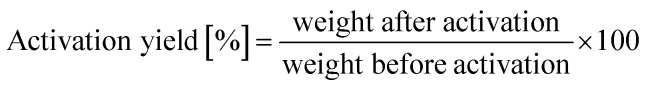


### 2.2 Adsorption measurements

Nitrogen adsorption–desorption isotherms at 77 K of the activated carbon prepared from C6 were obtained using a volumetric gas adsorption measurement device (Nova-e series; Anton Paar QuantaTec Inc., USA). Total pore volume and specific surface area were estimated through an *α*_S_-plot analysis^16^ of the N_2_ adsorption isotherms. Average pore width was calculated using the estimated total pore volume and specific surface area, assuming a slit-shaped pore. Pore size distribution was obtained using the quenched solid density functional theory (QSDFT) method.^17^ Micropore volume was calculated based on the size distribution of pores smaller than 2.0 nm.

Porosity was calculated as Porosity [cm^3^ cm^−3^] = total pore volume [cm^3^ g^−1^] × bulk density [g cm^−3^].

Where bulk density was estimated by packing each specimen into a cylinder with 1.6 mm inner diameter and 2.5 mm length.

Ethanol adsorption–desorption isotherms at 303 K were obtained using a commercial volumetric adsorption apparatus (Belsorp-max; MicrotracBEL Corp., Japan).

### 2.3 Morphological and chemical characterizations

We determined particle size using a scanning electron microscope (SEM, JSM-6700F; JEOL, Japan) and evaluated the mean size of 300 particles in SEM images of each sample. To observe microdomain structure, scanning tunneling microscope (STM, Agilent Technologies 5500 scanning probe microscope; Toyo Corporation, Japan) images were also acquired using its constant current mode (current: 0.3–1.2 nA, bias voltage: 0.1–1.5 V, and scan frequency: 1–2 Hz).

Raman spectra and elemental compositions are given in the ESI.†

## 3. Results and discussion

### 3.1 Pore structural characteristics

**Fig. 1:**
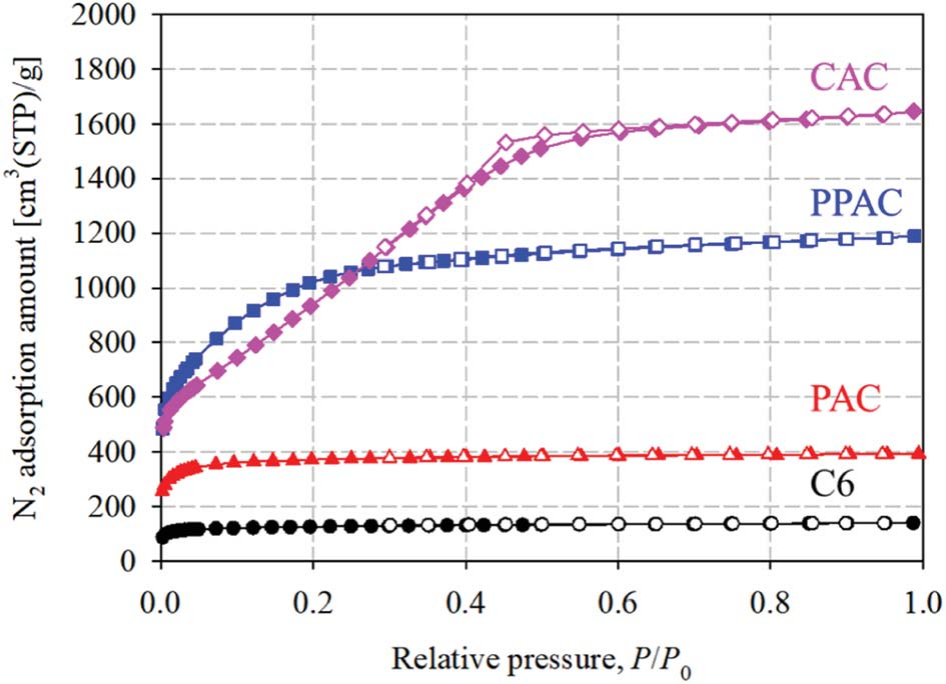
N_2_ adsorption–desorption isotherms at 77 K of starting carbonized material (C6), and the activated carbons derived *via* different activation methods. Solid and open symbols denote adsorption and desorption isotherms, respectively (circle: C6; triangle: PAC; square: PPAC; diamond: CAC).

Fig. 1 shows the N_2_ adsorption–desorption isotherms at 77 K of starting carbonized material (C6), and the activated carbons derived *via* different activation methods. PAC showed type I(a) isotherm, suggesting a presence of narrow micropores (of width <∼1 nm). PPAC and CAC showed type I(b) isotherms, though a small type H4 hysteresis loop was observed for CAC, according to the IUPAC classification.^18^ This suggests that PPAC and CAC have pore size distributions over a broader range including wider micropores and possibly narrow mesopores. The activation yield and pore structural parameters of each activated carbon are summarized in Table 2. The specific surface area and total pore volume of C6 were 570 m^2^ g^−1^ and 0.21 cm^3^ g^−1^, respectively. PAC showed an increased specific surface area of 1650 m^2^ g^−1^ and total pore volume of 0.60 cm^3^ g^−1^. PPAC showed a comparable activation yield to PAC, but a much higher degree of pore development, with a specific surface area of 3290 m^2^ g^−1^ and total pore volume of 1.78 cm^3^ g^−1^. Owing to chemical activation using KOH, CAC exhibited highly developed pores, with a specific surface area of 3010 m^2^ g^−1^, total pore volume of 2.45 cm^3^ g^−1^, and high activation yield of 31%. Chemically activated carbons are well known to exhibit higher activation yield and pore development than conventional physically activated carbons.^19^

**Table 2:** Activation yield and pore structural parameters of starting carbonized material and derived activated carbons

Sample	Activation pressure [MPa]	Activation yield [%]	Total pore volume [cm^3^ g^−1^]	Micropore volume [cm^3^ g^−1^]	Specific surface area [m^2^ g^−1^]	Average pore width [nm]
C6	—	—	0.21	0.18	570	0.73
PAC	0.1	13	0.60	0.56	1650	0.73
PPAC	1.0	12	1.78	1.39	3290	1.09
CAC	0.1	31	2.45	1.01	3010	1.65

Table 3 presents bulk densities and porosities calculated from total pore volume and bulk density. PPAC showed the highest porosity (0.69 cm^3^ cm^−3^), indicating effective pore development by pressurization. This high porosity was due to the high bulk density of PPAC (approximately twice that of CAC), although PPAC had a smaller total pore volume than CAC.

**Table 3:** Bulk density and porosity of starting carbonized material and derived activated carbons

Sample	Bulk density [g cm^−3^]	Porosity [cm^3^ cm^−3^]
C6	0.84	0.18
PAC	0.57	0.35
PPAC	0.39	0.69
CAC	0.21	0.51

**Fig. 2:**
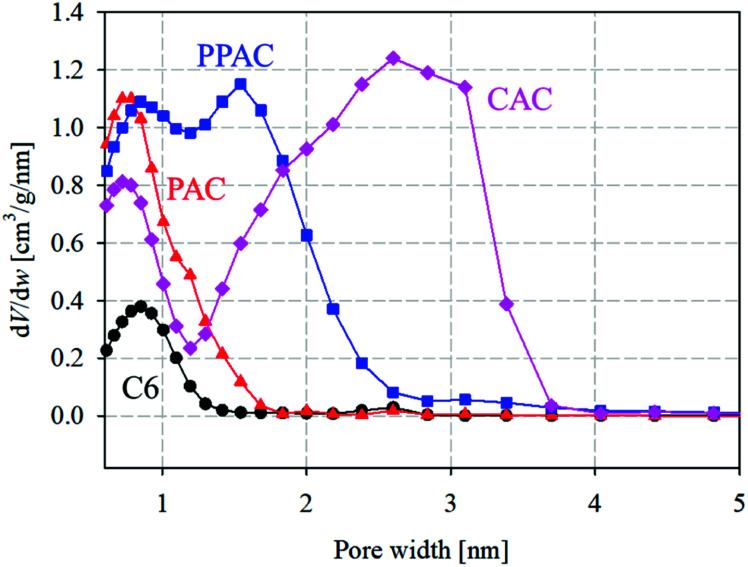
Pore size distributions for starting carbonized material and derived activated carbons (circle) C6; (triangle) PAC; (square) PPAC; (diamond) CAC.

Fig. 2 illustrates the pore size distributions calculated from the N_2_ adsorption isotherms at 77 K using the QSDFT method. Narrow micropores (<1.0 nm) in C6 were presumably voids formed during the thermal decomposition of raw phenol resin. PAC, prepared by the atmospheric pressure physical activation method, developed numerous narrow micropores with pore widths of <1.0 nm. Compared with PAC, CAC had fewer micropores smaller than 1.0 nm; however, it contained a large number of mesopores larger than 2.0 nm. In contrast, PPAC showed a characteristic bimodal pore size distribution, comprising narrow micropores smaller than 1.0 nm and wide micropores of approximately 1.6 nm. Pressurization enhanced the diffusibility of the activation agent (CO_2_ gas) into carbon particles and induced the development of inter-microdomain pores.^15^ Therefore, it was confirmed that the pressurized physical activation gave rise to the well-developed and unique pore structures between the atmospheric pressure physical activation and chemical activation methods.

### 3.2 Morphological characteristics

**Fig. 3:**
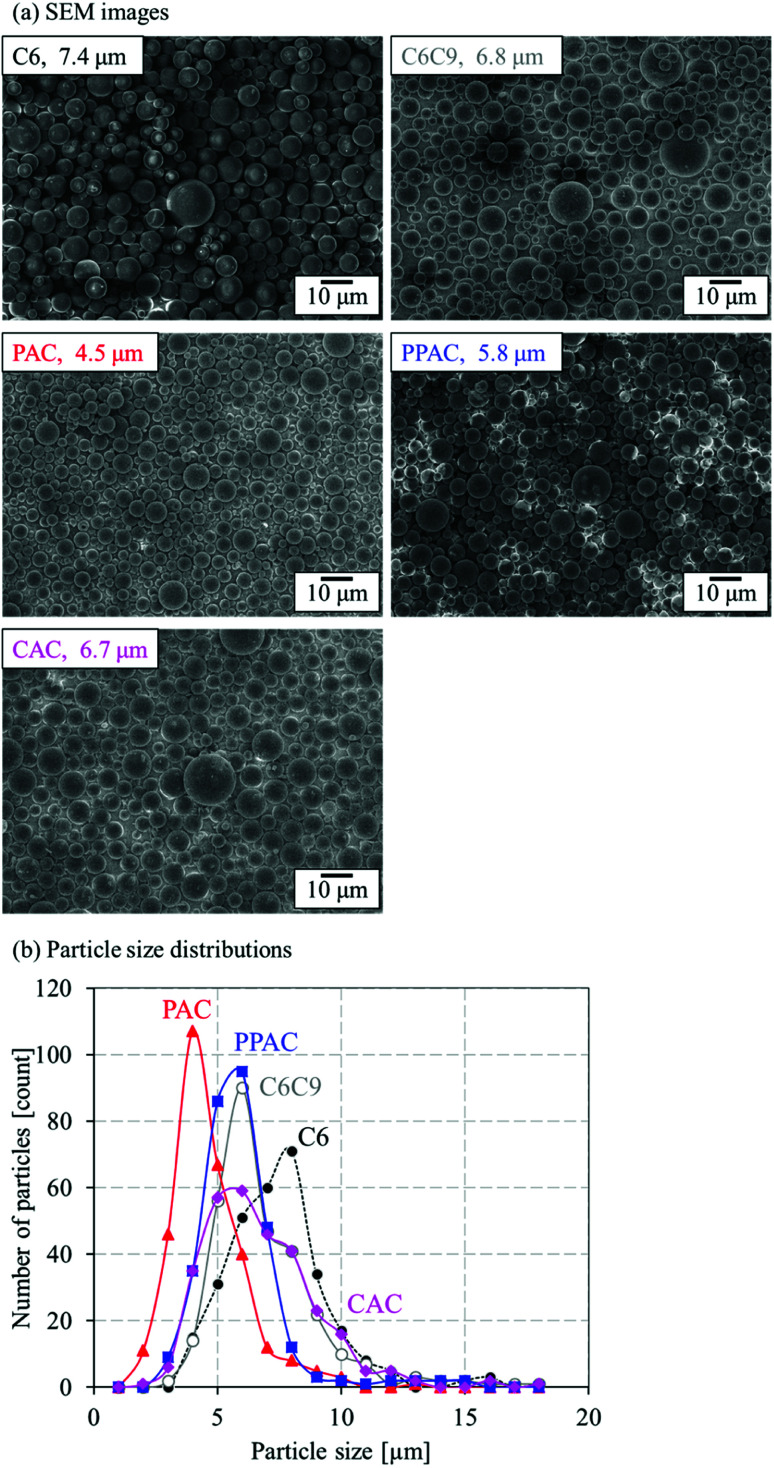
(a) SEM images and mean particle sizes and (b) particle size distributions of C6, C6C9, PAC, PPAC, and CAC.

Fig. 3 shows the SEM images and particle size distribution of C6, PAC, PPAC, and CAC. In addition, results of a heat-treated sample of C6 at 1173 K (900 °C) in N_2_ for 1 h (C6C9) are also shown in Fig. 3 for a comparison, because the activation process was carried out at 1173 K. To all samples, spherical shape of particle was maintained. Any treatment at 1173 K, that is, heat treatment in N_2_, atmospheric pressure and pressurized physical activations with CO_2_, and chemical activation with KOH, induced a decrease of particle size. The mean particle size of CAC almost agreed with that of C6C9 (6.7 and 6.8 μm, respectively). On the other hand, PAC showed a noticeable decrease of mean particle size to 4.5 μm. PPAC gave the mean particle size of 5.8 μm; intermediate between PAC and CAC. This suggests that the diffusibility of the activation agent into carbon particles for the pressurized physical activation was between the atmospheric pressure physical activation and chemical activation methods.

**Fig. 4:**
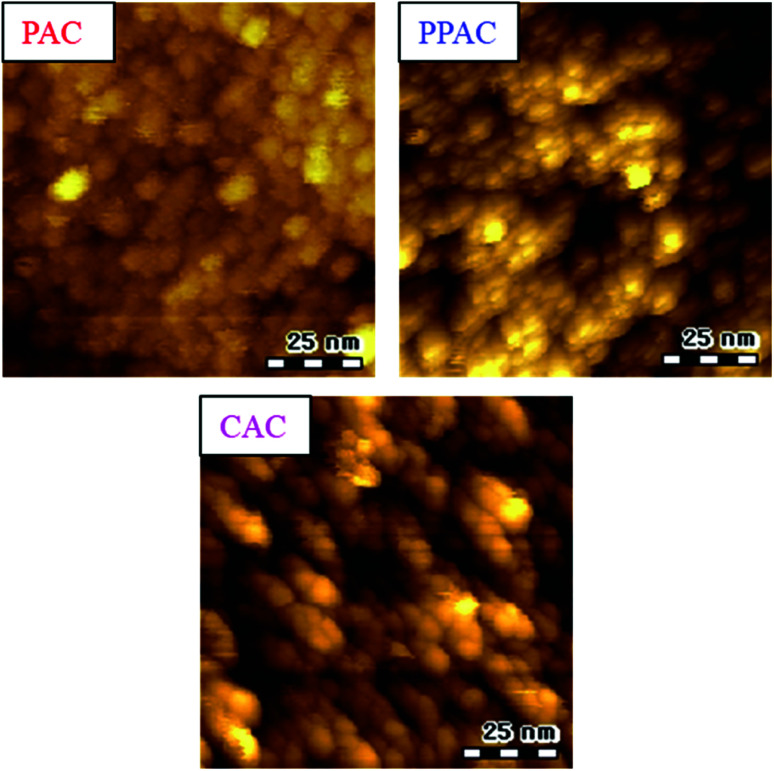
STM images of PAC, PPAC, and CAC.

STM images of all activated carbon samples are shown in Fig. 4. As reported in our previous works, inter-microdomain boundary suggests a development of inter-microdomain pores.^15,20^ PAC showed connected or unclear microdomain boundary. In contrast, PPAC and CAC showed clear inter-microdomain boundary. Moreover, CAC gave wider inter-microdomain gaps than PPAC. These results suggest that the wide pores including mesopores for CAC and the characteristic micropores of 1.6 nm for PPAC were originated from the inter-microdomain space.

### 3.3 Ethanol adsorption properties

**Fig. 5:**
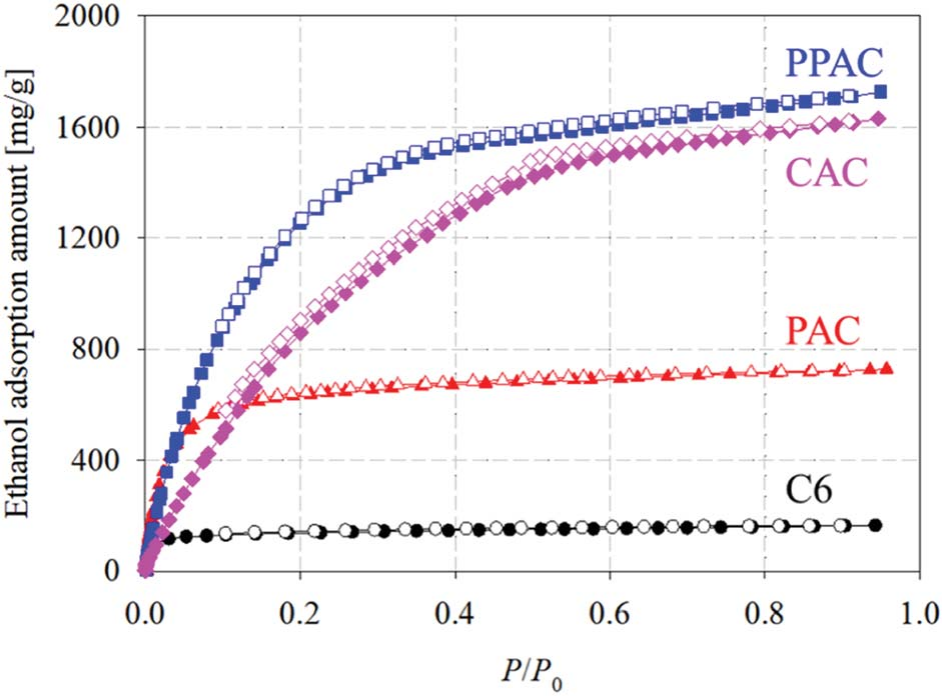
Ethanol adsorption–desorption isotherms at 303 K of starting carbonized material, and the derived activated carbons (circle: C6; triangle: PAC; square: PPAC; diamond: CAC).

**Fig. 6:**
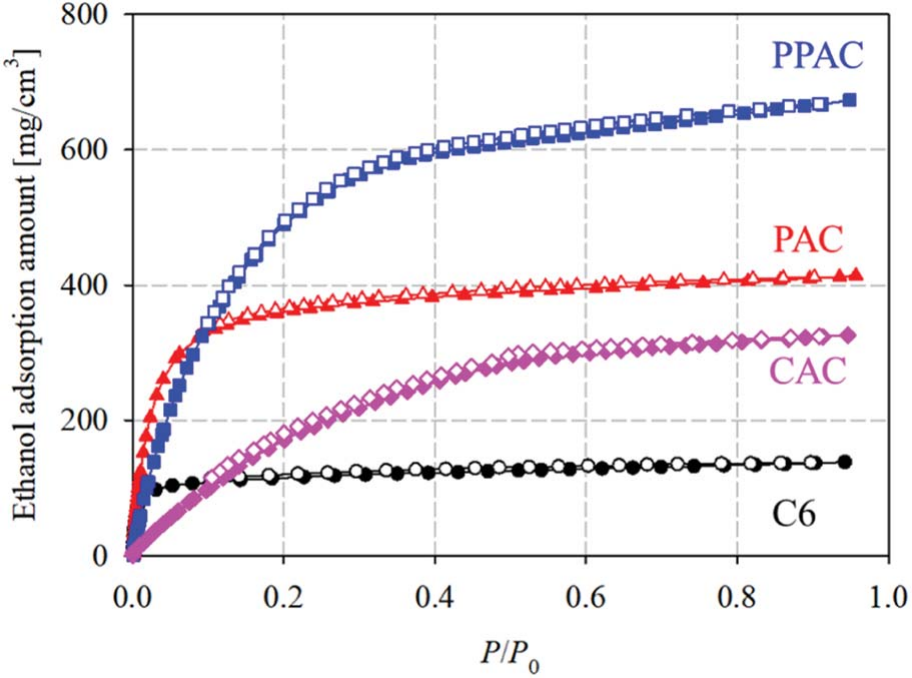
Adsorption–desorption isotherms on an adsorbent volume basis at 303 K of starting carbonized material, and the derived activated carbons (circle: C6; triangle: PAC; square: PPAC; diamond: CAC). The ethanol adsorption amounts on a volume basis were calculated from the ethanol adsorption amounts on a weight basis and the bulk density.

Fig. 5 shows ethanol adsorption–desorption isotherms measured at 303 K. Adsorption and desorption isotherms well overlapped each other for C6 and PAC, but PPAC and CAC showed small adsorption hysteresis loops at *P*/*P*_0_ > 0.4. From these isotherms, both the saturated (*P*/*P*_0_ = 0.95) and effective (*P*/*P*_0_ = 0.1–0.3) adsorption uptakes of ethanol were estimated (Table 4). For C6, the non-activated carbon, the saturated adsorption uptake was small (170 mg g^−1^), although the effective adsorption uptake was much smaller (10 mg g^−1^). According to the pore structural parameters (Table 2) and pore size distribution (Fig. 2), C6 possessed almost no pore structures that can contribute to the effective uptake of ethanol. The saturated uptake of ethanol by PAC was 730 mg g^−1^, which was significantly higher than that by C6 (170 mg g^−1^); however, the effective adsorption uptake was not so high enough (70 mg g^−1^). This was because the pores developed *via* conventional atmospheric pressure physical activation were mainly narrow micropores (<1.0 nm). Thus, PAC is not suitable as an adsorbent for AHP systems with ethanol refrigerant. Compared with PAC, CAC, with a high degree of pore development, showed over eight times higher effective ethanol uptake (580 mg g^−1^). PPAC achieved an effective ethanol uptake of 570 mg g^−1^, comparable with that of CAC. The characteristic pore size distribution of PPAC, with an optimal pore size of 1.6 nm,^14^ provided high ethanol uptake at *P*/*P*_0_ = 0.1–0.3 and thus showed the large effective adsorption uptake, despite its lower total pore volume than CAC. In addition, PPAC showed the highest effective ethanol uptake per unit volume (220 mg cm^−3^; Fig. 6 and Table 4). In contrast, the value for CAC was only 120 mg cm^−3^, because CAC contained a large number of wide pores, including mesopores, which contributed little to effective adsorption uptake. In other words, PPAC possessed “effective” pores. The superior volumetric ethanol adsorbability of PPAC suggests that it can improve AHP performance and reduce device size.

**Table 4:** Saturated and effective ethanol uptakes of starting carbonized material and derived activated carbons

Sample	Saturated adsorption amount of ethanol [mg g^−1^]	Effective adsorption amount of ethanol	
		On a weight basis [mg g^−1^]	On a volume basis [mg cm^−3^]
C6	170	10	8
PAC	730	70	40
PPAC	1730	570	220
CAC	1630	580	120

Furthermore, because of the post-treatment costs and expensive acid and alkali resistance equipment, chemical activation is much costlier than conventional atmospheric pressure physical activation. Although pressurized physical activation achieves a lower activation yield than chemical activation and requires a pressure-tight vessel, PPAC achieves a remarkably high effective ethanol uptake per unit volume; thus, it has potential as a relatively inexpensive, high-performance adsorbent for AHP systems with ethanol refrigerant.

## 4. Conclusions

In this study, we evaluated the applicability of pressurized physically activated carbon in AHP systems with ethanol refrigerant. The analyses of N_2_ adsorption–desorption isotherms at 77 K revealed that the carbon prepared *via* pressurized physical activation exhibited wide micropores of approximately 1.6 nm. Such micropores were not observed in activated carbons prepared *via* conventional atmospheric pressure physical activation or chemical activation. The pressurized physically activated carbon showed high effective ethanol uptake on a weight basis. The uptake was comparable with that of chemically activated carbon, despite the large difference in total pore volume between both carbon materials. Moreover, the pressurized physically activated carbon exhibited a remarkably high effective ethanol uptake per unit volume. Therefore, the pressurized physically activated carbon has great potential as an adsorbent for AHP systems with ethanol refrigerant.

As future works, to realize the AHP systems with PPAC–ethanol pairs, adsorption–regeneration cycle tests are planned to carry out for a confirmation of the long-term stability. Furthermore, as we have found that surface functional groups induced diffusional hindrance of ethanol molecules, giving rise to decreases of adsorption uptake and shortening of adsorption equilibrium time of ethanol,^21^ influence of surface modification of PPAC on the ethanol adsorbability will be also investigated.

## Author contributions

Hyeonseok Yi: investigation, validation, data curation, writing – original draft preparation. Koji Nakabayashi: validation. Seong-Ho Yoon: provision, project administration. Jin Miyawaki: conceptualization, methodology, validation, writing – reviewing and editing, project administration, supervision.

## Conflicts of interest

There are no conflicts of interest to declare.

## Supplementary Material

RA-012-D1RA08395C-s001
