# Supplementary material for: Study on the applicability of pressurized physically activated carbon as an adsorbent in adsorption heat pumps
Source: RSC Adv. 2022 Jan 19;12(5):2558–63. doi: 10.1039/d1ra08395c (PMC8979189; doi:10.1039/d1ra08395c)
Supplement: RA-012-D1RA08395C-s001 [file RA-012-D1RA08395C-s001.pdf]

Electronic Supplementary Material (ESI) for RSC Advances.
This journal is © The Royal Society of Chemistry 2022

Electronic supplementary information (ESI)

Study on the applicability of pressurized physically activated carbon as an adsorbent in adsorption heat pumps

Hyeonseok Yi, Koji Nakabayashi, Seong-Ho Yoon, Jin Miyawaki*

1. Raman spectroscopy

Raman spectra were obtained using a Raman spectrometer (NRS-3000, JASCO, Japan) employing a 532-nm Ar-ion laser as the excitation source. Intensity ratio of D band and G band, I_D/I_G, was calculated from the peak intensities at 1350 cm^{-1} (D band) and 1580 cm^{-1} (G band) in the Raman spectra.

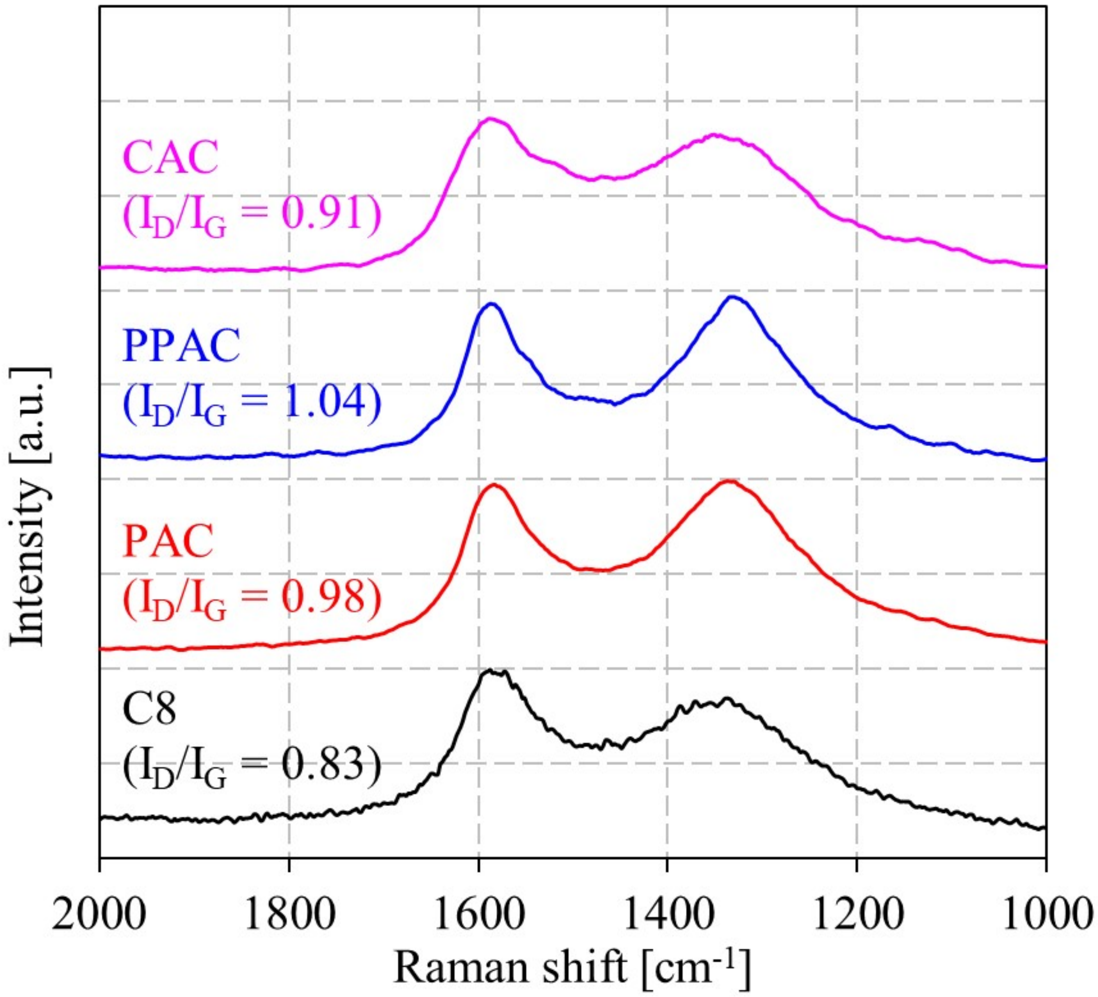

Figure S1. Raman spectra of starting carbonized material (C6), and the activated carbons derived via different activation methods (PAC, PPAC, and CAC).

2. Elemental composition

Elemental compositions of samples were analyzed using a CHN analyzer (MT-5, Yanako, Japan). The assay of O content ($O_{diff.}$) was defined by subtracting the sum of the contents of C, H, and N from 100%.

Table S1. Elemental composition of starting carbonized material (C6), and the activated carbons derived via different activation methods (PAC, PPAC, and CAC).

Sample	C [wt.%]	H [wt.%]	N [wt.%]	$O_{diff.}$ [wt.%][a]	Ash [wt.%]	$O_{diff.}$/C
C6	90.19	2.23	0.22	7.36	n.d.[b]	0.062
PAC	96.53	0.33	0.08	3.06	n.d.[b]	0.024
PAPC	93.66	0.34	0.09	5.91	n.d.[b]	0.047
CAC	95.15	0.02	0.14	4.69	n.d.[b]	0.037

[a] $O_{diff.}$ = 100 - (H + C + N).

[b] Not detected.
